# Research focus and emerging trends of the gut microbiome and infant: a bibliometric analysis from 2004 to 2024

**DOI:** 10.3389/fmicb.2024.1459867

**Published:** 2024-11-20

**Authors:** Ru Yang, Zeyao Shi, Yuan Li, Xi Huang, Yingxin Li, Xia Li, Qiong Chen, Yanling Hu, Xiaowen Li

**Affiliations:** ^1^Department of Neonatology Nursing, West China Second University Hospital, Sichuan University, Chengdu, China; ^2^Key Laboratory of Birth Defects and Related Diseases of Women and Children (Sichuan University), Ministry of Education, Chengdu, China

**Keywords:** infant, gut microbiome, atopic dermatitis, necrotizing enterocolitis, microbiome transmission, VOSviewer, CiteSpace, visualization

## Abstract

**Background:**

Over the past two decades, gut microbiota has demonstrated unprecedented potential in human diseases and health. The gut microbiota in early life is crucial for later health outcomes. This study aims to reveal the knowledge collaboration network, research hotspots, and explore the emerging trends in the fields of infant and gut microbiome using bibliometric analysis.

**Method:**

We searched the literature on infant and gut microbiome in the Web of Science Core Collection (WOSCC) database from 2004 to 2024. CiteSpace V (version: 6.3.R1) and VOSview (version: 1.6.20) were used to display the top authors, journals, institutions, countries, authors, keywords, co-cited articles, and potential trends.

**Results:**

A total of 9,899 documents were retrieved from the Web of Science Core Collection. The United States, China, and Italy were the three most productive countries with 3,163, 1,510, and 660 publications. The University of California System was the most prolific institution (524 publications). Van Sinderen, Douwe from University College Cork of Ireland was the most impactful author. Many studies have focused on atopic dermatitis (AD), necrotizing enterocolitis (NEC), as well as the immune mechanisms and microbial treatments for these diseases, such as probiotic strains mixtures and human milk oligosaccharides (HMOs). The mother-to-infant microbiome transmission, chain fatty acids, and butyrate maybe the emerging trends.

**Conclusion:**

This study provided an overview of the knowledge structure of infant and gut microbiome, as well as a reference for future research.

## 1 Background

In recent years, with the advancements in intensive care medicine, the birth and survival rates of high-risk infants have increased significantly, such as preterm infants, low-birth-weight infants, and infants with various congenital defects (Walani, 2020; Cao et al., 2022; Kang et al., 2023). Compared to healthy full-term infants, the surviving high-risk infants face both short and long-term problems. The potential lifelong impact lasts into their adolescence and adulthood, taking an enormous burden on individuals, families, health systems, and society (Mericq et al., 2017; Crump et al., 2020). The gut microbiota plays an important role in nutrition absorption, metabolism, immune system establishment, and neurodevelopment of infants (Houghteling and Walker, 2015; Yang et al., 2016). The composition and dysbiosis of gut microbiome in early life are associated with long-term health outcomes such as obesity, type 1 diabetes, atopic dermatitis (AD), food allergy, allergic asthma, childhood respiratory diseases, and neurocognitive development (Vu et al., 2021; Kostic et al., 2015; Petersen et al., 2021; Tamana et al., 2021; Stokholm et al., 2020; Alcazar et al., 2022; Donald and Finlay, 2023; Depner et al., 2020).

The infant’s gut microbiome displays distinct from the adults in microbial functions and composition (Barker-Tejeda et al., 2024). During the first months of life, the community of infants exhibited considerable heterogeneity and instability compared with adults (Yang et al., 2024). Multiple factors influence the development and establishment of the infant’s gut microbiota. Vertical microbiome transmission from mother to infant during birth and in early life, may elicit the potential immune stimulation and induce the metabolic and developmental transcriptional programs (Jašarević and Bale, 2019; Wampach et al., 2018; Koren et al., 2024; Duranti et al., 2017). Other maternal factors such as maternal microbiome, inflammation, diet, obesity, diabetes, maternal stress and mood impact the assembly of the infant gut microbiome (Grech et al., 2021; Vatanen et al., 2022; Prentice et al., 2023; Galley et al., 2023; Pärnänen et al., 2018). Delivery mode influences the initial colonization of the gut microbiota, shaping the first gut microbes of the newborn and continuing to influence up to 4 years after birth (Reyman et al., 2019; Mitchell et al., 2020; Fouhy et al., 2019). After birth, infants are continuously exposed to multiple microbiome communities (Yassour et al., 2018; Hildebrand et al., 2021; Brooks et al., 2017), and factors such as gestational age, antibiotic exposure, breastfeeding, lifestyles, and the introduction of solid food shape the infant gut microbiome (Reyman et al., 2022; Olm et al., 2022; Davis et al., 2022). Despite the multifactorial influences on infant gut microbial succession, the communities of infant gut microbiome followed predictable patterns in the early stages of life (Stewart et al., 2018). The community structure of infant microbiota matures during early life and gradually develops slowly toward an adult-like profile (Reyman et al., 2019; Wang et al., 2021).

Otherwise, previous studies have shown probiotics can improve the gut microbiome in preterm infants (Beck et al., 2022). Evidence revealed that probiotic supplementation modifies the gut microbiome in preterm infants, leading to a bifidobacterium-dominated microbiome community (Alcon-Giner et al., 2020). The probiotics mixture containing strains of *bifidobacterium* species accelerates the microbiome maturation in extremely preterm infants to term-like microbiome with higher stability and species interconnectivity (Samara et al., 2022). Besides, probiotic is an effective measure to prevent necrotizing enterocolitis (NEC) and neonatal sepsis for preterm infants (Aceti et al., 2017; Mercer and Arrieta, 2023). NEC is one of the leading causes of neonatal mortality, is characterized by the acute onset of patchy necrosis throughout the intestine, leading to systemic sepsis (Duess et al., 2023). The development of NEC is attributed to immature intestinal development, intestinal barrier dysfunction, gut microbiota dysbiosis, and an imbalance of Toll-like receptor-mediated intestinal inflammation in the intestinal mucosa of preterm infants (Duess et al., 2023; Hackam and Sodhi, 2018). Transfer of the microbiome isolated from human breast milk-fed preterm infants’ fecal samples to germ-free mice protects against pathogen infection and is associated with a significant increase in intestinal Th17 cells, which significantly attenuates NEC-like injuries induced in neonatal mice (Colliou et al., 2017). Besides, human milk oligosaccharides (HMOs) are the third most abundant constituent in human milk (HM) following lactose and lipids, which play an important role in preventing NEC and late-onset sepsis (LOS), modulating intestinal epithelial cell response, improving the gastrointestinal barrier/immune function, and improving brain development/cognitive function (Vatanen et al., 2022; Dinleyici et al., 2023; Zhang et al., 2022; Kassai and de Vos, 2024; Kiely et al., 2023; Salminen et al., 2020).

Despite the considerable progress made in this area, there is no research providing a comprehensive and exhaustive analysis of the current literature on the gut microbiome and infant. The study aims to provide an overview of global literature on gut microbiota and infants from 2000 to 2024 using bibliometric methods. Collaboration networks are demonstrated through countries, institutions, and authors, while keyword analysis will indicate the current research hotspots in the field and possible future research trends. Citespace and VOSviewer are methods of bibliometric literature, freely available computer software for constructing and viewing bibliometric maps (van Eck and Waltman, 2010; Chen, 2004). Bibliometrics describe or show the published information and the related metadata using statistics, such as the journals, countries, authors, keywords, etc. (Ninkov et al., 2022).

## 2 Methods

We searched the literature on infant and gut microbiome in the Web of Science Core Collection (WOSCC) database using the Medical Subject Headings (MeSH) and keywords from 2004 to 2024. We use the term “infant,” “Gastrointestinal Microbiome” and all of the hyponyms to retrieve relevant literature. The search strategy was shown as follows: topic = (gut OR intestinal OR gastrointestinal OR Gastric OR Enteric) AND topic = (microbiota* OR microbiome* OR flora OR microflora OR Microbial OR bacteria) AND topic = (infant* OR newborn* OR neonat* OR Prematur* OR preterm). Then, we used CiteSpace V (version: 6.3.R1) and VOSview (version: 1.6.20) to display the top authors, journals, institutions, countries, authors, keywords, co-cited articles, and emerging trends.

## 3 Results

### 3.1 Analysis of annual publications

**Figure 1:**
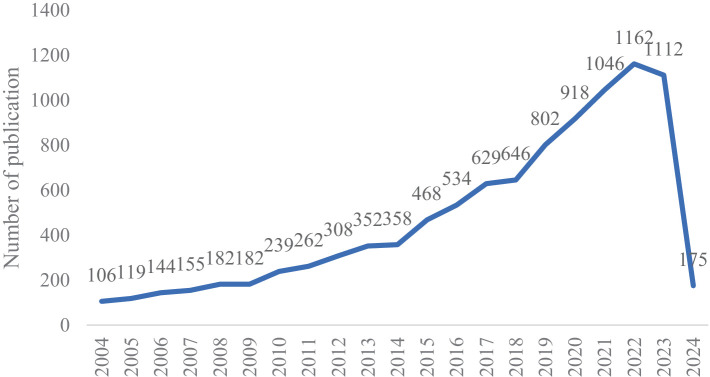
Number of annual publications and growth trends on infant and gut microbiome from 2004 to 2024.

We retrieved a total of 10,479 documents, removing duplicates and irrelevant documents to end up with 9,899 documents. Publication was limited to “articles” and “reviews.” As shown in Figure 1, the number of publications on infant and gut microbiome has been a steady increase over the years. Since 2021, the field has experienced a sustained peak with more than 1,000 publications per year. Compared to 2004 (106 publications), the number of publications in 2021 (1,162 publications) had a nearly more than sevenfold increase.

### 3.2 Map of the collaborative network of country/region, and institution

**Figure 2:**
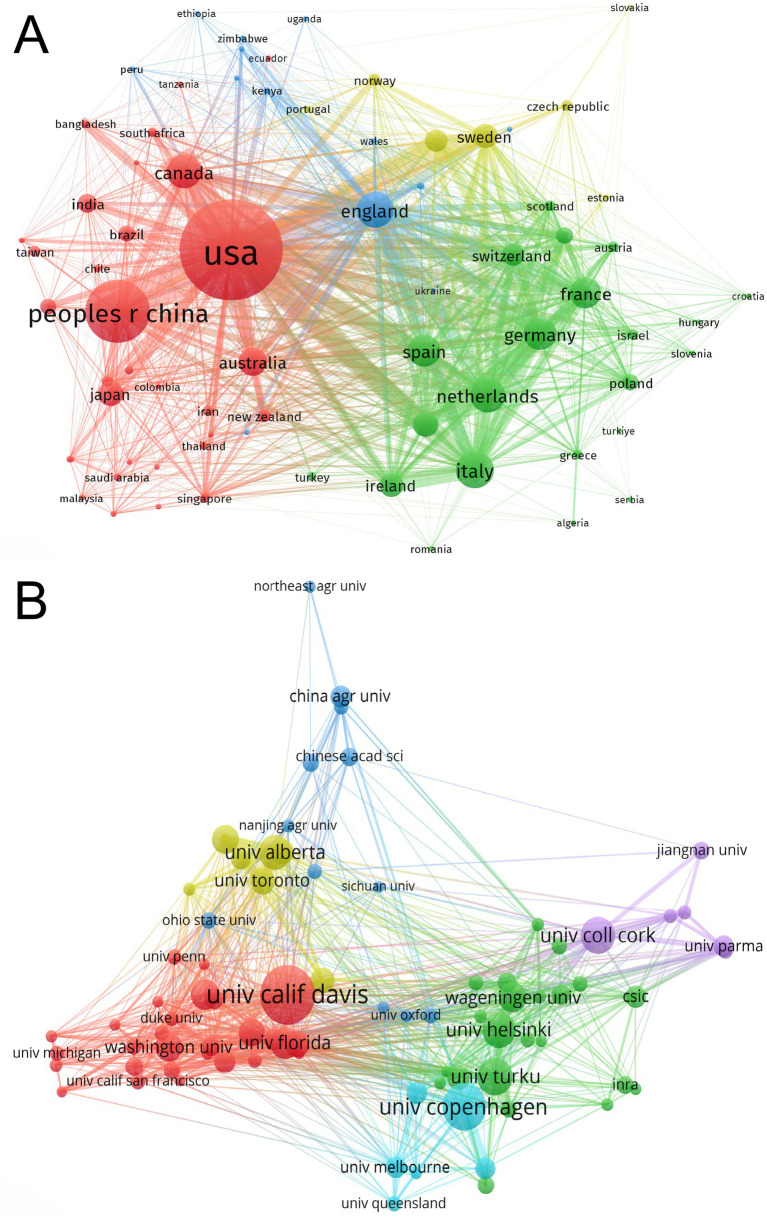
Maps of collaborative networks of countries/regions (A) and institutions (B) in the field of infant and gut microbiome (2004–2024). This map was produced by VOSviewer. The threshold for the collaborative network of country/regions was set at a minimum of 15 publications, and 73 countries/regions reached this threshold. The threshold for institutional collaborative networks was set at a minimum of 47 publications, and 81 institutions reached this threshold. The colors indicate the separate groups of collaborators.

The top 10 country/region and institution in terms of number of publications in the field of infant and gut microbiome are presented in Tables 1, 2. The United States, China, and Italy were the three most productive countries with 3,163, 1,510, and 660 publications, respectively. The USA contributed to most of the research in the field, with a total of five institutions ranking among the top 10 in terms of publication count. The maps of the co-authorship are visualized as the collaborative networks of countries/regions and institutions in Figures 2A,B. Three main collaborative groups led by United States, China, and Australia were identified in Figure 2A. Several centers were formed with these three countries, with the University of California Davis as the main research location, with strong ties to institutions such as University of Florida, Washington University, Duke University, and University of Pennsylvania. There are also other several institutions developed centers, such as the University of Copenhagen, University of Turku, University of Helsinki, Wageningen University & Research, University College Cork, University of Alberta, and University of Toronto. China, as a developing country, has also contributed a great deal of scholarship to this field, such as Nanjing Medical University, China Agricultural University, Chinese Academy of Medical Sciences, Nanjing Agricultural University, and Sichuan University. Chinese institutions have collaborated with institutions from other countries to a certain extent, but the level of cooperation is not yet sufficiently close.

**Table 1:** Top 10 countries/regions in terms of number of publications in the field of infant and gut microbiome (2004–2024).

Rank	Country	Publications	Centrality
1	USA	3,163	0.18
2	China	1,510	0.01
3	Italy	660	0.09
4	England	629	0.13
5	Netherlands	586	0.07
6	Canada	579	0.12
7	Germany	529	0.1
8	Spain	515	0.08
9	France	494	0.15
10	Australia	432	0.08

**Table 2:** Top 10 institutions in terms of number of publications in the field of infant and gut microbiome (2004–2024).

Rank	Institution	Country	Publications	Centrality
1	University of California System	USA	524	0.11
2	Harvard University	USA	280	0.1
3	University College Cork	Ireland	266	0.05
4	University of California Davis	USA	249	0.08
5	Consejo Superior de Investigaciones Cientificas (CSIC)	Spain	217	0.05
6	Wageningen University and Research	Netherlands	200	0.06
7	University of Copenhagen	Denmark	195	0.21
8	INRAE	France	178	0.11
9	State University System of Florida	USA	173	0.06
10	Institut National de la Sante et de la Recherche Medicale (Inserm)	USA	171	0.05

### 3.3 The most top 10 journals

The documents in this field were published in 1,899 different journals. Table 3 shows the 10 journals publishing the highest number of documents in this field. Nutrients, Frontiers in Microbiology, Plos One, Scientific Reports, and Journal of Pediatric Gastroenterology and Nutrition were the 5 journals that published the highest number of publications. All the top 10 journals were in Q2 and Q1.

**Table 3:** Top 10 journals in terms of number of publications in the field of infant and gut microbiome (2004–2024).

Rank	Journal	Publications	%	IF	JCR
1	Nutrients	352	3.328	5.9	Q1
2	Frontiers in Microbiology	291	2.751	5.2	Q2
3	Plos One	259	2.448	3.7	Q1
4	Scientific Reports	225	2.127	4.6	Q2
5	Journal of Pediatric Gastroenterology and Nutrition	165	1.56	2.9	Q2
6	Pediatric Research	162	1.531	3.6	Q1
7	Frontiers in Immunology	156	1.475	7.3	Q1
8	Microorganisms	140	1.324	4.5	Q2
9	Gut Microbes	139	1.314	12.1	Q1
10	Frontiers in Pediatrics	128	1.21	2.6	Q2

### 3.4 Map of the collaborative network of authors

**Figure 3:**
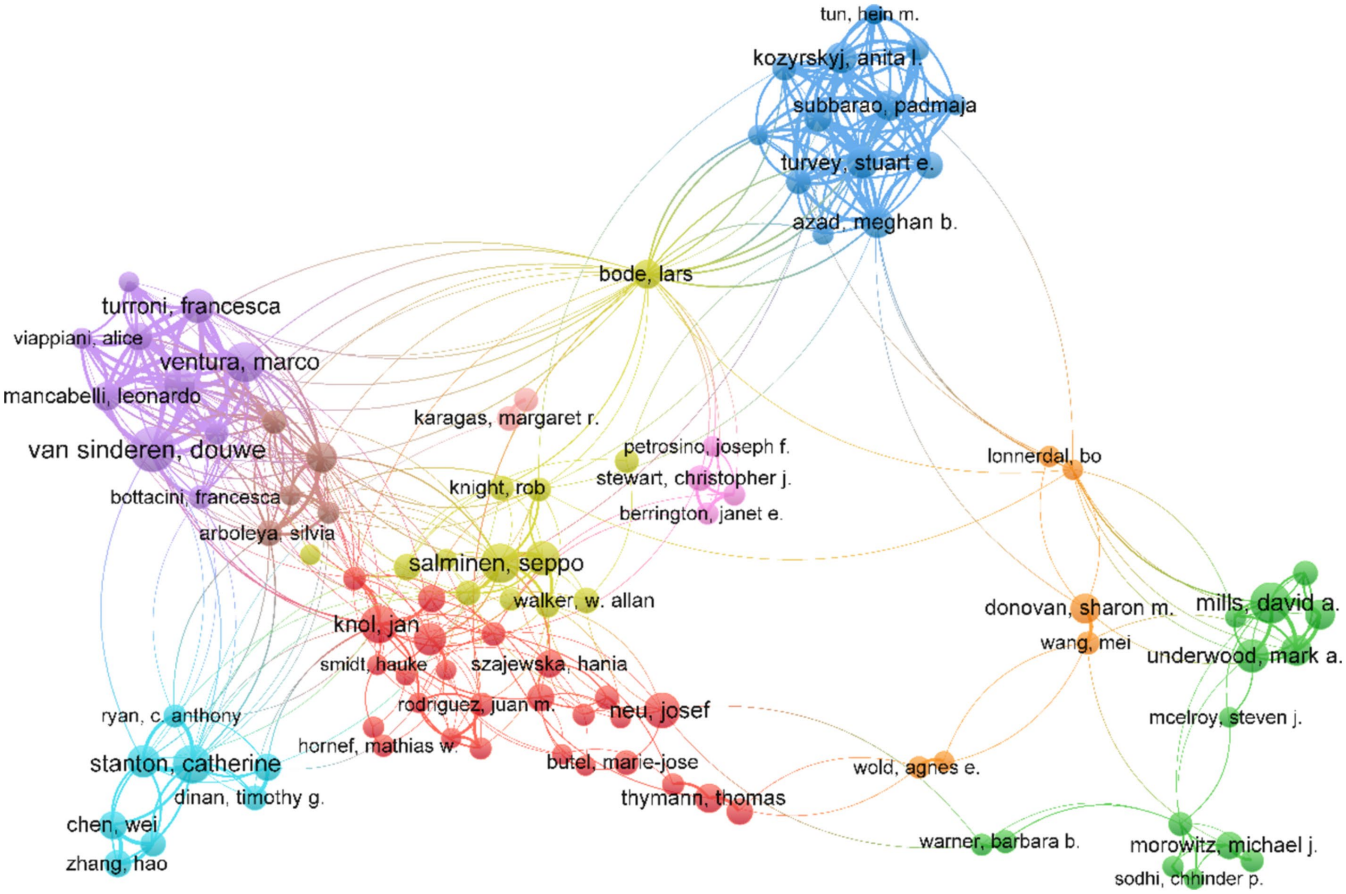
Map of the collaborative network of authors in the field of infant and gut microbiome (2004–2024). This map was produced by VOSviewer. The threshold for the collaborative network of authors was set at a minimum of 20 publications, and 107 authors reached this threshold. The colors indicate the separate groups of collaborators.

Table 4 shows the top 10 authors publishing the highest number of documents in this field. Van Sinderen, Douwe from University College Cork of Ireland was the most impactful author, followed by Ventura, Marco from the University of Parma, and Salminen, Seppo from the University of Turku. Several main collaborative networks were identified, led by Mills David A, Salminen Seppo, Stanton Catherine, Ventura Marco, and Van Sinderen, Douwe (Figure 3).

**Table 4:** Top 10 authors in terms of number of publications in the field of infant and gut microbiome (2004–2024).

Rank	Author	Institution	Country	Publication	Centrality
1	Van sinderen, Douwe	University College Cork	Ireland	92	0.05
2	Ventura, Marco	University of Parma	Italy	78	0.01
3	Salminen, Seppo	University of Turku	Finland	73	0.02
4	Mills, David A	University of California	USA	71	0.01
5	Knol, Jan	Wageningen University/Danone Nutricia Research	Netherlands	63	0.05
6	Stanton, Catherine	University College Cork	Ireland	62	0.03
7	Milani, Christian	University of Parma, Parma	Italy	52	0.01
8	Turvey, Stuart E	University of British Columbia	Canada	47	0
9	Turroni, Francesca	University of Parma	Italy	46	0.01
10	Neu, Josef	University of Florida	USA	40	0.02

### 3.5 The most top 10 cited articles

Co-citation refers to the situation when two studies are cited together by another study at the same time, which indicates a significant relationship between studies (Jiang et al., 2024). A highly cited article can serve as an important landmark in a particular field. It plays a crucial role in a subject area by making significant contributions to the existing knowledge base and exerting influence on subsequent research (Chen, 2004). Highly cited literature not only reflects the current focal points of research but also provides indications of potential future research trends.

The top 10 publications receiving the highest number of citations in this field are shown in Table 5. The article most cited was Stewart, C.J entitled: “Temporal development of the gut microbiome in early childhood from the TEDDY study” (Stewart et al., 2018). As we can also find from analysis of the cited literature, in the last 20 years, researchers have focused on the development trajectory and factors influencing the gut microbiome in early infant life, such as breast milk, cesarean section (CS), and antibiotics. Recently, mother-to-child microbial transmission has become an emerging focus. In addition, with the rapid development of DNA sequencing and bioinformatics technology, researchers have explored the microbiome more deeply. QIIME 2 is a microbiome bioinformatics platform that provides multiple interactive visualization tools for the latest generation of sequence quality control, taxonomic assignments, and phylogenetic insertion for different sequencing platforms (Bolyen et al., 2019).

**Table 5:** Top 10 publications with the highest number of citations in the field of infant and gut microbiome (2004–2024).

Cited by	Author(s), year, country	Journal	Research type	Title	Method
442	Stewart et al. (2018), USA	Nature	Article	Temporal development of the gut microbiome in early childhood from the TEDDY study	16S rRNA + Metagenomic analysis
435	Bäckhed et al. (2015), Copenhagen	Cell host and microbe	Article	Dynamics and Stabilization of the Human Gut Microbiome during the First Year of Life	Metagenomic analysis
316	Ferretti et al. (2018), Italy and Germany	Cell host and microbe	Article	Mother-to-Infant Microbial Transmission from Different Body Sites Shapes the Developing Infant Gut Microbiome	Metagenomic analysis
296	Milani et al. (2017), Italy	Microbiology and molecular biology review	Review	The First Microbial Colonizers of the Human Gut: Composition, Activities, and Health Implications of the Infant Gut Microbiota	
293	Pannaraj et al. (2017), USA	JAMA pediatrics	Article	Association Between Breast Milk Bacterial Communities and Establishment and Development of the Infant Gut Microbiome	16S rRNA
280	Yatsunenko et al. (2012), USA	Nature	Article	Human gut microbiome viewed across age and geography	16S rRNA
267	Aagaard et al. (2014), USA	Science translational medicine	Article	The placenta harbors a unique microbiome	16S rRNA + Metagenomic analysis
246	Shao et al. (2019), UK	Nature	Article	Stunted microbiota and opportunistic pathogen colonization in cesarean-section birth	Metagenomic analysis
243	Bokulich et al. (2016), USA	Science translational medicine	Article	Antibiotics, birth mode, and diet shape microbiome maturation during early life	16S rRNA
237	Bolyen et al. (2019), USA	Nature biotechnology	Review	Reproducible, interactive, scalable and extensible microbiome data science using QIIME 2	

### 3.6 Keywords and hotpots for infant and gut microbiome

**Figure 4:**
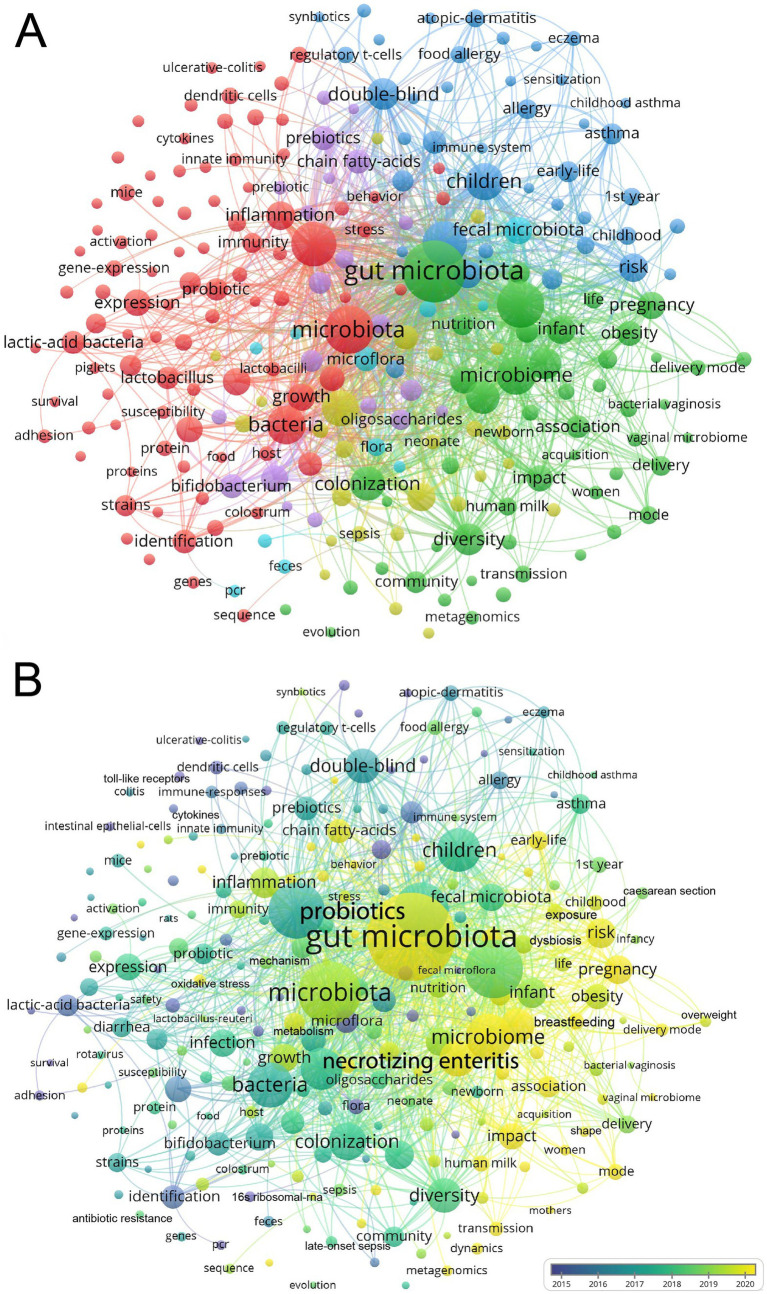
Network visualization cluster map of keywords in the field of infant and microbiome from 2004 to 2024. This visualized map of keywords was developed when the minimum-term occurrences were placed at least 72 times. Two hundred and forty two terms reached this threshold out of 22,971 in this field, which were divided into six clusters (A). The colors of the map range from purple to yellow representing the year in which the article was published (B).

We used VOSviewer to conduct a keyword visualization analysis, selecting keywords that appeared at least 72 times. Out of the 22,971 keywords, 242 keywords met this threshold. The high-frequency keywords of published articles were clustered into 6 categories (Figure 4A). Cluster 1 and Cluster 2 mainly focused on “innate immunity”, “inflammation”, “expression”, and “infection”. Cluster 3 focused on allergic diseases and the long-time influences, such as “atopic dermatitis”, “food allergy”, “asthma”, “childhood”, “long term consumption”. Cluster 4 focused on “preterm infants”, “birth weight infants”, “necrotizing enterocolitis”, “human milk oligosaccharides”, “bifidobacterium”, “lactobacilli”, “probiotics”, “supplementation”. Cluster 5 and Cluster 6 focused on “delivery mode”, “cesarean section”, “vaginal microbiome”, “transmission”, “exposure”, “shape”, “young child, and”, “obesity”.

VOSviewer color-codes keywords based on their appearance over time, from blue to yellow, representing when the article appeared from the earlier to the later (Figure 4B). The first trend for gut microbiome transmission from mother to infant, including the delivery mode and vaginal microbiome. The second trend was “chain fatty acids,” “oxidative stress,” “butyrate,” and “metabolism” (Figure 4B; Supplementary Figure S1).

## 4 Discussion

Advances in medical technology have improved the survival rates of high-risk infants such as preterm and low birth weight infants, and the quality of long-term survival of these children is of concern. Over the past two decades, the field of microbiology has undergone significant transformations. The widespread use of metagenomes sequencing has allowed us to provide rich insights into the composition and functional exploration of gut microbiome. This exploration has also help us to revealed the full map of infant gut microbiome, including fungi, viruses, and other ecosystems.

We conducted a literature analysis and visualization in the field of infant and gut microbiome. The publication of scientific literature reflects the level of scholarly attention to a particular field. Over the past 20 years, there has been a continuous increase in publications in this field. Since 2021, it has entered a phase of rapid development.

Researches on infant and gut microbiome are primarily concentrated in developed countries. The United States has made the most prominent contributions in this field, followed by China, Italy, the United Kingdom, and the Netherlands. China, as a developing country, has also made significant academic contributions to this field. This may be attributed to China’s policies and social conditions. China implemented the “two-child policy” in 2016 and the “three-child policy” in 2021. With the implementation of these policies, the number of elderly pregnant women in China is increasing continuously. Additionally, in recent years, there has been widespread use of assisted reproductive technology and advancements in intensive care medical technology. All of these factors have contributed to the increase in the birth rates and survival rates of high-risk infants in China. As the gut microbiome plays a crucial role in human health and disease. Chinese scholars pay more attention to the field of infant and gut microbiome.

From the cluster analysis, we could find that the immune, allergic diseases, NEC, HMOs, and CS were the focus of the field. Moreover, the mother-to-infant microbiome transmission, the chain fatty acids, and butyrate maybe the emerging trends.

There are many risk factors for allergic diseases, including delivery mode, diet, urban living, antibiotic exposure, and the early gut microbiome community (Donald and Finlay, 2023; Chu et al., 2017; Kirjavainen et al., 2019; Patrick et al., 2020; Lee-Sarwar et al., 2023; Galazzo et al., 2020). Impaired gut microbiota maturation may be mediating the development of all pediatric allergic diseases, encompassing the alteration of a core group of species, functional pathways, and metabolic imbalance (Hoskinson et al., 2023; Wopereis et al., 2014). Breastfeeding enrichment of *Bifidobacterium longum subsp. infantis* is associated with reduced antibiotic-associated asthma risk (Dai et al., 2023). The mixed strain of *Lactobacillus* and *Bifidobacterium* can effectively reduce the incidence of eczema in infants under 3 years old (Sun et al., 2021). Oral administration of *Lactobacillus rhamnosus* showed a therapeutic effect in children with moderate AD (Jeong et al., 2020). A meta-analysis that included 5,406 children with AD showed that probiotic strains mixtures, such as *Lactobacillus* and *Bifidobacterium* subspecies, reduced the risk of AD for at least up to 7 years (Tan-Lim et al., 2021).

NEC is one of the leading causes of neonatal mortality, particularly in preterm and extremely low birth weight infants. The overall mortality rate in newborns with confirmed with NEC was approximately 23.5% (Jones and Hall, 2020). There is currently no specific treatment for NEC. Surviving infants experience severe long-term complications, including intestinal failure, short bowel syndrome, growth restriction, and impaired neurodevelopment, such as cerebral palsy, intraventricular hemorrhage, and periventricular leukomalacia (Matei et al., 2020; Wang et al., 2024). The pathogenesis of NEC remains obscure, NEC is an intestinal inflammatory disorder (Cho et al., 2020). Most studies support the crucial role of Toll-like receptor 4 (TLR4) activation in NEC development (Hackam and Sodhi, 2018; Werts et al., 2020). Mice with enterocyte-specific deletion of TLR4 were protected from NEC (Sodhi et al., 2012), and TLR4 expression is very high in the intestinal mucosa of preterm infants compared to term intestines (Niño et al., 2016). Additionally, altered gut microbiota composition is associated with NEC in preterm infants. The gut microbiome of preterm infants altered several days prior to the development of NEC, which characterized by an increase in the abundance of Proteobacteria, such as *Klebsiella*, and *Enterocolitis*, and a decrease in *Firmicutes* (Pammi et al., 2017; Olm et al., 2019; Ward et al., 2016). The dysbiosis microbiome invades the immature intestine of preterm infants and interacts with the overexpressed TLR4 in the intestinal epithelial cells, triggering pro-inflammatory responses in the intestinal mucosa and underlying endothelial cells, leading to the development of NEC (Hackam and Sodhi, 2022). HM had a significant protective effect on NEC (Miller et al., 2018). Exclusive HM–Based Diet reduces the incidence of NEC from 16.7 to 6.9% (Hair et al., 2016). Breast milk can activate aryl hydrocarbon receptor (AHR) in the neonatal intestine and reduce TLR4 signaling in the neonatal intestinal epithelium (Lu et al., 2021). HMOs may attenuate intestinal NEC-associated inflammation through interacting with the lipopolysaccharide (LPS) binding site on TLR4 (Sodhi et al., 2021). HM is the predominant source of Immunoglobulin A (IgA) for preterm infants in the first month and the relative decrease of IgA-bound bacteria is associated with the development of NEC (Gopalakrishna et al., 2019).

In addition, Group B streptococcus (GBS), a *β*-hemolytic Gram-positive encapsulated bacterium that commonly colonizes the vagina and intestines of adults, is a major cause of neonatal sepsis and meningitis (Gonçalves et al., 2022; Lohrmann et al., 2023). Neonatal meningitis is a devastating disease associated with high mortality and long-term neurological developmental deficits, with serious economic and health burden on individuals, families, and society (Mynarek et al., 2024; Horváth-Puhó et al., 2021). Neonates are exposed to multiple microbiome sources after birth, and the neonatal gut is susceptible to GBS colonization during the perinatal and postnatal periods. GBS can result from maternal colonization and vertical transmission from the mother or subsequent intestinal seeding (Tazi et al., 2019). The immature gut microbiome of infants is associated with decreased resistance GBS colonization, which favors translocation across the gut barrier (Travier et al., 2021). The main strategies for preventing the neonatal meningitis are based on intrapartum antibiotic prophylaxis (IAP). IAP has significantly reduced the incidence of early-onset disease (EOD), it is not effective against of late-onset disease (LOD) (Braye et al., 2018). The use of IAP disrupts the development of maternal and neonatal gut microbiota, which may be associated with chronic metabolic diseases later in life (Garcia, 2021; Azad et al., 2016). Although GBS may be transmitted to infants through breast milk, the beneficial components in human milk may inhibit GBS colonization in the infant gut. HMOs have antimicrobial and anti-biofilm activity against GBS, and IgA in breast milk binds to GBS and reduces the risk of LOD (Mejia et al., 2022; Moore et al., 2022; Dangor et al., 2020). Probiotics, such as Lactobacilli have antagonistic activities against GBS (Bnfaga et al., 2023; Hanson et al., 2022), and oral administration of Lactobacilli reduces the GBS colonization at 35–37 weeks of gestation (Menichini et al., 2022). Furthermore, maternal vaccination is likely to be a cost-effective intervention to protect fetuses and newborns from GBS invasion (Hanson et al., 2022).

Multiple factors influence and alter the infant gut microbiome, and more research has focused on how to restore or improve the perturbed infant gut microbiome. Gut microbiome therapies include probiotics, fecal microbiota transplantation (FMT), and vaginal seeding. Probiotics, in particular, are widely employed to regulate and improve infant gut microbiome (Mercer and Arrieta, 2023). However, there are still some controversies regarding the effectiveness of probiotics in applications. This may be mainly due to the lack of standards, the genetic heterogeneity of the cohort, and the diversity of probiotics (Xiang et al., 2023).

A recent technique to restore disrupted gut microbes in CS infants is maternal microbiome seeding, including vaginal seeding and FMT (Hourigan et al., 2022; Korpela and de Vos, 2022). The purpose of maternal microbiota seeding is to restore the gut microbiome of CS infants closer to that of vaginally delivered infants, thereby reducing the incidence of chronic inflammatory diseases associated with CS infants. Vaginal seeding involves wiping over the infant body with sterile gauze filled with the mother’s vaginal microbiome or orally administering the mother’s vaginal microbial solution (Song et al., 2021; Dominguez-Bello et al., 2016; Wilson et al., 2021). FMT commonly used for the treatment of recurrent *Clostridium difficile* infections and other gastrointestinal disorders, and now being explored in infant (Korpela et al., 2020). Although observational studies suggest that vaginal seeding and FMT may temporarily, partially restore infant gut microbiome, there are no randomized controlled trials showing that mother-infant microbial seeding alters the infant microbiome. More research is needed to understand the long-term effects of these interventions on infants.

Despite the growing interest in mother-infant microbiome seeding, controversies persist regarding safety, principles, and regulations. Further studies, large-scale, well-designed randomized controlled trials, are essential to determine the effectiveness of these interventions in altering infant gut microbiome and improving long-term health outcomes.

There were some limitations in our study. Firstly, we analyzed the publications only researched from the WoSCC, publications of other databases related to our topic may retrieved incompletely. The WoSCC is the most influential scientific database and is widely used for bibliometric analysis. Furthermore, the visualization analysis was limited to published academic literature, thus it may potentially ignore relevant research from unpublished or non-academic institutions.

## 5 Conclusion

We reveal the knowledge collaboration network, research hotspots, and explore the emerging trends in the fields of infant and gut microbiome. The United States, China, and Italy were the three most productive countries. Many studies have focused on AD, and NEC, as well as the immune mechanisms and microbial treatments for these diseases, such as probiotic strains mixtures (bifidobacteria, actinomycetes) and HMOs. And recently, the mother-to-infant microbiome transmission, chain fatty acids, and butyrate maybe the emerging trends. This study provided an overview of the knowledge structure of infant and gut microbiome, as well as a reference for future research.

## Data Availability

The raw data supporting the conclusions of this article will be made available by the authors, without undue reservation.
