# Supplementary material for: Research focus and emerging trends of the gut microbiome and infant: a bibliometric analysis from 2004 to 2024
Source: Front Microbiol. 2024 Nov 20;15:1459867. doi: 10.3389/fmicb.2024.1459867 (PMC11615055; doi:10.3389/fmicb.2024.1459867)
Supplement: Supplementary file 2 [file Supplementary_file_2.docx]

**Supplementary Figure**


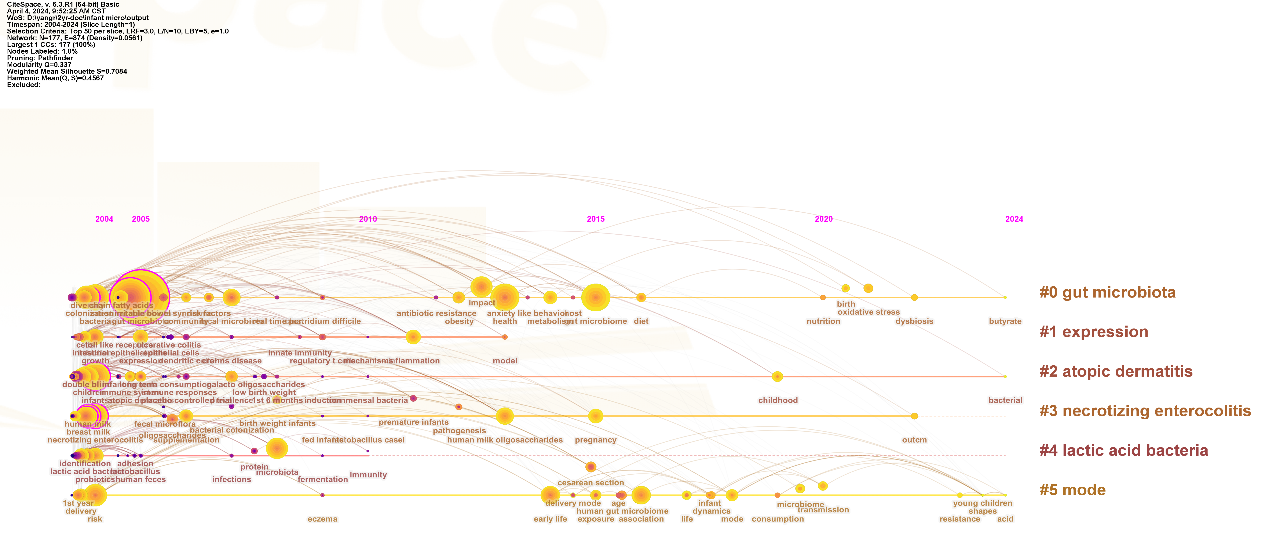


**Figure S1.** Timeline view map of reference co-citation analysis generated by CiteSpace on infant and gut microbiome from 2004 to 2024.
